# Good citizens, perfect patients, and family reputation: Stigma and prolonged isolation in people with drug-resistant tuberculosis in Vietnam

**DOI:** 10.1371/journal.pgph.0000681

**Published:** 2022-06-22

**Authors:** Lisa Redwood, Greg J. Fox, Thu Anh Nguyen, Sarah Bernarys, Paul Mason, Van Anh Vu, Viet Nhung Nguyen, Ellen M. H. Mitchell

**Affiliations:** 1 The Faculty of Medicine and Health, The University of Sydney, Central Clinical School, The University of Sydney, Camperdown, New South Wales, Australia; 2 The Woolcock Institute of Medical Research, Ba Dinh District, Hanoi, Vietnam; 3 The University of Sydney, School of Public Health, The University of Sydney, Camperdown, New South Wales, Australia; 4 Taronga Conservation Society Australia, Sydney, Australia; 5 The University of Sydney, School of Education and Social Work, The University of Sydney, Camperdown New South Wales, Australia; 6 National Tuberculosis Program, Hanoi, Vietnam; 7 Department of Public Health, Tropical Infectious Disease Group, Institute for Tropical Medicine, Antwerp, Belgium; PLOS: Public Library of Science, UNITED STATES

## Abstract

Stigma and isolation are common in people with tuberculosis (TB). Social isolation contributes to reduced health outcomes and TB treatment adherence. Stigma and the drivers of isolation in people with Drug-Resistant (DR)-TB may include modifiable advice and practices of family and Health Care Workers (HCW). This study aimed to understand the drivers of isolation and stigma from the perspective of people with DR-TB in Vietnam. A greater understanding of stigma and isolation is important to identify and balance patients’ needs and disease transmission risk. In-depth interviews were conducted with 12 people with DR-TB and seven HCWs who care for people with DR-TB in two provinces in Vietnam. Interviews were audio-recorded, transcribed verbatim and translated to English. Data collection and analysis were conducted simultaneously. The data were then analysed using a thematic framework approach. Stigma and extended isolation were common experiences among people with DR-TB. To mitigate stigma, people with DR-TB used the local term ‘lao lực’ to describe their condition to others which is believed to be a less infectious and less stigmatising type of TB. This study identified that although HCW informed people with DR-TB of when they were no longer infectious and isolation was no longer required, their infection control advice was not always consistent. Despite knowing they were no longer infectious, most people with DR-TB continued to self-isolate to minimise the perceived repercussions of societal stigma, to protect their ‘thể diện’ (honour, prestige, reputation), and eliminate all risk of transmitting DR-TB to their family. This study identified three interconnected drivers of self-isolation in Vietnam, including fear of infecting others, fear of stigmatization, and to protect family reputation. TB control programmes need to better understand the social aspects of DR-TB to enable them to better support patients. Educating HCW to provide evidence-based infection control advice is vital.

## 1. Introduction

The incidence of tuberculosis (TB), a mycobacterial infectious disease, poses a public health concern with the rise of drug-resistant (DR) strains [1]. While improved treatment regimens and the availability of monitored treatment delivery options in high TB burden countries have increased treatment completion rates for TB, the treatment completion rate for people with DR-TB and multidrug-resistant (MDR)-TB remains low [2]. In Vietnam, the treatment completion rate for MDR-TB was 69% in 2019, compared to drug-susceptible TB which was 91% [2]. A systematic review identified the common social stressors for people with MDR-TB to be stigma (self/internalised, anticipated and enacted), self-isolation, lack of social support, separation from family, loneliness, and family and community rejection [3]. These stressors are often not addressed alongside TB care and can contribute to reduced treatment adherence in people with DR-TB [4–6].

Isolation is multidimensional, complex and can outlast the initiating condition [7,8]. As with stigma, there are several attributes of isolation including who is imposing the isolation (i.e., a person’s own decision to self-isolate, or as a result of the behaviour of others); the type of isolation (social isolation or physical isolation); and the duration of isolation (e.g., short–months or long–years) [9]. During the early stages of TB treatment, physical isolation is appropriate and necessary for infection control purposes, ideally in a negative pressure room [10]. This is required until the mycobacteria have been suppressed by an adequate treatment regimen, typically after the first two weeks of treatment for drug-susceptible TB, and after one to three months of treatment for DR-TB [11,12]. However, globally, people with TB and DR-TB are often isolated for extended periods of time from their family and friends [3]. In the absence of clear clinical advice for the duration of physical isolation at home and a supportive and understanding community, people with TB can become trapped without a predictable end to their social and physical isolation, which can endure considerably longer than medically justified [13,14].

Social Isolation can increase morbidity and reduce treatment adherence in people with DR-TB. Humans are social beings and thrive on engagement with others. Social isolation can be defined as–“the objective lack of interaction with others or the wider community” [15]. Isolation reduces the amount of social support received and can be detrimental to the health of people, including those with DR-TB [3,15]. The COVID-19 pandemic which was declared in 2020 has exposed everyone to social isolation to reduce disease transmission. This extended isolation has seen a reduction in life satisfaction and psychological wellbeing resulting in increased rates of depression and anxiety across all age groups [16,17]. In people with DR-TB and with other infectious diseases, social isolation and loneliness can also harm medication adherence [18–20]. Despite the significant impact that isolation can have on the morbidity of people with DR-TB, through isolation itself and reduction in treatment adherence, there remains limited evidence of the drivers of the isolation and clear infection control advice provided to patients [13]. Therefore, it is important to collect in-depth data surrounding the drivers and the impact of stigma and isolation in high DR-TB burden countries to learn how to better support and empower people with DR-TB throughout their treatment journey and beyond.

This paper examines the stigma and isolation experienced, the advice provided from their health providers and the drivers of isolation in people with DR-TB in Vietnam.

## 2. Methods

The thematic analysis research method was used in this study [21]. The study design included in-depth-interviews (IDI) with people with DR-TB and health care workers (HCW) working in DR-TB departments in Vietnam. Some elements of grounded theory were used in the data collection process [22]. The coding and preliminary data analysis occurred throughout data collection and the developing themes were explored in future interviews [22].

### 2.1. Study setting

Vietnam is a Southeast Asian country located on the South China Sea with a population of 96 million [23]. In 2019, 3.3% of new TB cases and 17.7% of previously treated TB cases were resistant to the antibiotic rifampicin, the cornerstone of current TB therapy (rifampicin-resistant TB (RR-TB)) or both rifampicin and isoniazid (MDR-TB) [2]. Vietnam is included in the top 30 high MDR-TB burden countries, with an incidence rate of 8.8 (95% CI 5.5–13) per 100,000 population in 2019 [2]. Treatment for RR/MDR-TB is provided through public Programmatic Management of Drug-resistant TB (PMDT) centres, primarily through lung hospitals. This study was set in Hanoi and Thanh Hoa provinces in northern Vietnam. These sites were chosen as they have a high prevalence of TB and they provide perspectives from both urban and more rural locations [24]. In 2018, Northern Vietnam had a prevalence of 262 (95%CI 180–380) per 100,000 for bacteriologically confirmed TB [24]. Thanh Hoa is located 160 kilometres south of Hanoi.

### 2.2. Sampling and inclusion criteria

Maximum variation purposive sampling method was used [25]. This study aimed to interview a heterogeneous, purposive sample of adult people with DR-TB. We aimed to maximize variations in characteristics that can affect illness experience and stigma, such as gender, age, treatment duration and treatment adherence. To enable the triangulation of information, people with DR-TB and HCW responsible for treating patients with DR-TB were recruited. All people with DR-TB were required to have bacteriologically confirmed pulmonary DR-TB (defined as having a positive GeneXpert MTB/RIF test for both rifampicin resistance and *M*. *tuberculosis*, or another drug susceptibility test confirming rifampicin resistance) and be currently receiving antibiotics for TB from the National Tuberculosis Program. People with DR-TB were also required to be aged 18 years or older and have completed at least three months of treatment to reduce the risk of disease transmission to the interviewer. Participants were excluded from the study if their usual residence was outside of the participating provinces, they had an unconfirmed diagnosis or people who were currently incarcerated.

HCWs were required to be 18 years or older and have worked in a DR-TB facility for the previous 12 months. We aimed to interview senior HCW, including the head DR-TB doctor and head nurse in the department, as they would have a better understanding of how DR-TB stigma affects themselves and their patients.

### 2.3. Recruitment

People with DR-TB were recruited by a trained qualitative Vietnamese researcher with the aid of DR-TB department staff at the Hanoi Lung Hospital and Thanh Hoa Lung Hospital. The DR-TB department staff were informed of the inclusion criteria and sampling method. Their role was to provide a summary of the study and invite people with DR-TB to participate. The researcher would then meet with the person to explain the study in detail, go through the Patient Information Statement and answer any questions. Prior to the interview, written informed consent was obtained. The HCW participants were invited to participate in the study by a qualitative Vietnamese researcher.

### 2.4. Study participants

The study participants comprised of people with DR-TB and HCW from the DR-TB departments in Hanoi Lung Hospital and Thanh Hoa Lung Hospital. The sample size was not known until the study progressed and theoretical data saturation was achieved. The sample size of people with DR-TB and HCW recruited for this study was guided by the saturation criterion, in which the recruitment of new study participants ceased when no new relevant themes or categories were emerging [22]. Saturation was reached after interviewing 12 people with DR-TB and seven HCW.

### 2.5. Data collection

The interviews were conducted by the local researcher in a private room located in the hospital and were scheduled on their regular hospital visit to make it more convenient for the participants. Data were collected between November 2017 and February 2018. One author (LR) attended several initial interviews to observe the environment and interactions between the people with DR-TB and others. Study participants received approximately US$5 to compensate for their time based on the incentives usually given to research participants in Vietnam. Each participant had one IDI lasting between 45 and 75 minutes. Sociodemographic data were obtained through observation and by asking the participant.

The researcher followed flexible topic guides containing open-ended questions. The topic guides were derived from a literature review on stigma and isolation in people with TB and DR-TB and in discussion with the local researcher team and the authors. They contained introductory questions followed by more exploratory questions to help establish a rapport with the participant and to encourage openness, reflection, and honesty. Interviews were participant-led, with the researcher following up on the answers and probing for meaning. The interview guide was continually updated to allow further exploration of the emerging concepts [22] (S1 Text).

The interviewer took field notes after each interview to record observations, behaviours or other relevant information that may have been missed in the interviews. All interviews were audio recorded using a mobile phone, manually transcribed, and translated to English verbatim for preliminary hand-coding, analysis, and theory development.

### 2.6. Data analysis

A thematic analysis was used with some elements of grounded theory [21,22,26]. This study used a continuous comparative approach to analyse the data throughout data collection, which is an element of grounded theory [22]. Each interview was coded and compared to the previously analysed transcripts and the field notes. Existing codes were then refined, and new codes were developed based on the new interview and how it compared to the previous interviews.

A thematic analysis was then used to derive the main codes/themes both inductively and deductively. Themes were coded and recoded in collaboration with the authors (S2 Text). The themes were triangulated for commonalities and differences by comparing them to the field notes and HCW interviews. A thematic network was then developed by arranging and re-arranging the themes in a logical hierarchical manner. A framework approach was subsequently used to transform the thematic network and interview transcripts into a matrix in an Excel spreadsheet to enable the comparison of themes and a summary of the themes for each participant [26].

### 2.7. Trustworthiness and rigor

Credibility, transferability, dependability, and confirmability were used to evaluate the trustworthiness of this study [27]. Creditability was enhanced by the triangulation of the data, thematic analysis, and the collaboration between three qualitative researchers and the local research team on the research process and analysis. Transferability is demonstrated through purposive sampling, the transcription of IDI verbatim and a dense description of the methodology. Dependability and confirmability were achieved by the triangulation of data, the coding and recoding of the data and reviewing the emerging analysis with peer checks between the authors.

### 2.8. Researcher participant relationship

Before the study commenced, the researchers examined their personal bias and stigma toward TB and MDR-TB by describing and discussing the anticipated outcomes of the interviews. To minimise the potential bias in study participants, a local researcher with previous experience working with people with TB, conducted the interviews, rather that HCWs. During the recruitment process, the participants were informed that the interviewer is external from the health care system and anything they said would not interfere with their TB care. Despite aiming to locate a quiet, private location for the interview, this was sometimes unachievable for hospitalised participants and HCW who were very busy. During some of these interviews a nurse or family member would interrupt the interview, which may have made the participant more reluctant to truthfully share their experiences.

### 2.9. Research ethics

The human research ethics committee at the University of Sydney (2017/730) and the Institutional Review Board in Vietnam approved this study (No: 73/12/CT-HDKH-DD). The staff in the participating Lung Hospitals briefly explained the study to eligible people with DR-TB and invited them to participate in the study. The staff explained that the study was being conducted externally to the hospital and their decision to participate would not impact their care. No timeframe given to the eligible population to decide. The staff informed the local researcher of an interested participant. The local researcher then met the interested participant, explained the study in detail and went through the Patient Information Statement. The interview was then either conducted on the same day, or a time suitable for the participant. Written informed consent was obtained from each participant by the local researcher prior to the interview. Interviews took place in a private and quite space. The local researcher had not worked at either of the participating hospitals.

## 3. Results and discussion

The sample included a diverse mix of people with DR-TB, including five people currently receiving inpatient care and seven people receiving outpatient care. The duration of treatment that the patient had received at the time of the interview ranged from three to 17 months. We recruited five females and seven males aged between 28 and 67. Two patients with a history of non-adherence to their prescribed TB treatment regimen were interviewed, as this group is often negatively impacted by stigmatization [28]. We interviewed seven HCW, of which five worked at the DR-TB department at Hanoi Lung Hospital and two worked at Thanh Hoa Lung Hospital. All HCWs had been working in the DR-TB department for over one year. Four of the HCW were nurses and three were doctors (Table 1).

**Table 1:** Sociodemographic information of the study participants.

		People with DR-TB (n = 12)		Health Care Workers (n = 7)	
		n	(%)	n	(%)
Age (mean)		40		-	
Gender	Female	5	(41.7)	5	(71.4)
	Male	7	(58.3)	2	(28.6)
Province	Hanoi	10	(83.3)	5	(71.4)
	Thanh Hoa	2	(16.7)	2	(28.6)
Patient setting	Inpatient	5	(41.7)	-	
	Outpatient	7	(58.3)	-	
Healthcare worker	Doctor	-		3	(42.9)
	Nurse	-		4	(57.1)
Length of treatment at time of interview (months)		6.9		-	

DR-TB = drug-resistant tuberculosis.

The study explored stigma and isolation in people with DR-TB in Vietnam. Isolation post-infectious period was common in households with the person with DR-TB sleeping separately and restricting bowl and utensils used as if fomite transmission were possible (section 3.1). The extent of social isolation varied between participants. Our analysis revealed that people with DR-TB are informed by their doctors of when they are no longer infectious and their subsequent low risk of transmitting DR-TB. However, the infection control advice given is not always consistent (section 3.2). This study identified three interconnected drivers of physical and social isolation in Vietnam including fear of infecting others, fear of stigma, and protecting their self and familial reputation (known as ‘thể diện’) (section 3.3). Lastly, we discovered how a local term for TB was used to mitigate stigma in Vietnam (section 3.4).

### 3.1. Stigma and isolation in people with DR-TB Vietnam

People with DR-TB in Vietnam were aware of the public stigma surrounding TB and often chose not to disclose their condition to others to avoid their anticipated stigma.

*“I think in Vietnam people do not dare to share information*. *From my experience and observation*, *nobody can be confident to tell others that they have TB*. *Because people do not have good knowledge about DR-TB*, *and they do not have a proper attitude about DR-TB patients… I just want to be treated like a normal person who can wear [a] mask while talking but others’ attitude is not right with stigma*, *being pointed at by others” (Woman*, *single*, *under 30*, *Hanoi-4 months of treatment*)

People with DR-TB in Vietnam also experienced isolation from their family and friends. Family members were afraid of contracting DR-TB and encouraged isolation.

*“Out-clinic patients go home*, *and their family members fear being infected as this is a communicable disease*. *So*, *they are expected to stay in their room and have no communication*, *the patients feel frustrated and stuck*. *They are sick but nobody wants to get close to them*. *That makes them feel down” (Woman*, *nurse*, *Hanoi*)

These findings are similar to those found in other settings. Disclose of TB status varies widely and is influenced by many factors. A recent study in Uganda, found that 30% of people with TB chose not to disclose their status to their household members, while in South Africa, 65% of people with TB did not disclose their status to people outside their household [29,30]. Family and community rejection and social disconnection are identified as common social stressors in people with TB and MDR-TB [31].

Several study participants were socially excluded, i.e., were asked to not attend social events or were avoided by their friends. The participants often reported feeling alone and felt like giving up at least once during their treatment. A young lab technician recounted when her two roommates, who were doctors, moved out after she confided her diagnosis with them and had ceased contact.

*“When I knew that I got TB*, *I informed the people around me*, *including my two roommates who are doctors*. *I was very sad and talked to my roommates*. *They both moved out right on that day*. *I have been alone in the house since then*. *I felt so lonely*, *nobody was beside me… (Crying) then I was so upset*, *and it was like I was in a depression… when I knew that I had DR-TB*, *I just wanted to die*, *(crying)…I did*, *honestly…because I am still young*, *my career is developing…my best friends left me” (Woman*, *single*, *under 30*, *Hanoi-4 months of treatment*)

The HCWs were able to provide many examples of the different ways that people with DR-TB were isolated. They recounted the common concerns of the patients’ families such as infection control, which led to prolonged separation. They also shared the most extreme cases that they had witnessed in their career, including divorce, or patients’ having their children taken away from them by family members.

*“Some female patients with DR-TB here are divorced*. *Her husband left after she was infected*. *She talked to me and cried a lot*. *Her husband even did not let her keep their child*. *She was not allowed to keep their child because she was infected with DR-TB*. *It is terrible for her*. *She is at risk of developing XDR-TB*. *I asked her to come for treatment*, *but she did not want to*. *It sounds like she’s dissatisfied and fed up with it” (Woman*, *Doctor*, *Hanoi*)

Despite the many stories of isolation and social exclusion by people with DR-TB, not all participants experienced it. Some people reported that they were not socially isolated, and their life was mostly unchanged following their diagnosis. The man below describes that during the month that he was isolated at home, he would continue to be invited over to his neighbour’s house for tea.

*“Q*: *Did you visit and spend time with them [your neighbours]*?

*A*: *Yes… I went to their house*. *They invited me*, *told me to come to have a drink*. *Each time I was at home*, *he told me to come to drink water*, *so I accepted… they sell iced tea and other herbal drinks*. *They asked me to come to drink water for fun*. *Because I only stayed at home that month [after diagnosis]*.*” (Man*, *married*, *under 60*, *Hanoi-7 months of treatment*)

Every participant with DR-TB had experienced some form of stigma and medically unnecessary isolation, often resulting in distress. These findings mirrors and extends previous work which demonstrated the isolation in people with TB and DR-TB. Similar expressions of isolation have been described in people with TB in Ethiopia by sleeping and eating separately from their families [32]. While in Nigeria a common concern among people with MDR-TB was being separated from their partner [33]. In Vietnam, there was a tendency to conflate the potentially protective proscriptions against shared air and the largely symbolic proscriptions against share utensils.

### 3.2. Health care workers advice

HCWs are at the forefront of the patient’s questions and anxieties surrounding their DR-TB care, treatment, and fears of transmission. The doctors and nurses reported having a good relationship with their patients. They followed them up regularly and provided advice and support when their patients returned home. Some HCWs also provided their patients with their personal phone number. One nurse also described how some patients used the hospital as a social outlet, as they felt less stigmatized and were able to connect and bond with people going through the same experiences [note: wearing a face mask at the lung hospitals is mandatory in Vietnam, regardless of infectiousness].

*“Many patients come back to the hospital because they cannot talk to anyone at home because they are stigmatized*. *It [isolation and stigma] is not too bad with families who understand TB*. *They still eat together and do not isolate the patient*. *But there are many families*, *like when neighbors say "he has TB*, *do not have contact with him"… in here*, *nobody distinguishes between who has TB and who does not have TB*.*” (Woman*, *nurse*, *Hanoi*)

Most patients acknowledged having been informed by their doctor of their negative test results and when their risk of transmission was low.

“*The doctor said when I left the hospital that the bacteria were negative… The smear and culture results were negative from the third and fourth month*, *so the ability to spread it [DR-TB] is relatively low” (Man*, *married*, *under 40*, *Hanoi-6 months of treatment*)

However, the infection control advice given was variable and not always strictly evidence based. One doctor recommended wearing masks and isolating for six months, defined as sleeping separately but eating together. While another doctor advised patients that once they are discharged from the hospital, they are no longer infectious, and therefore no infection control measures were required. People with DR-TB can be hospitalised for one to two weeks whilst they undergo testing or longer, depending on symptom severity and other comorbidities [34].

*“A*: *I remind them [the patients] to wear masks regularly and take medicine on time*. *I also advised them to isolate themselves for 6 months*.

*Q*: *Do you recommend eating separately from their family*?

*A*: *No*, *sleeping alone should last for 6 months but eating separately is not recommended*. *However*, *many patients worry and want to protect their family members*, *so they have separate meals and sleep separately until the end of treatment*. *Some even send their children to grandparents*.*” (Man*, *doctor*, *Hanoi*)

                                 ----

*“There is discrimination among patients’ families*. *They ask us many questions about eating or share a room with the patient*. *Of course*, *we have to counsel them…they (family members) don’t want to eat and stay in the same house with the patient…some patients came back home and are forced to live alone… Their family asked them to eat and sleep separately…We have to counsel them that although DR-TB is contagious*, *when a patient is allowed to come back home*, *it means since then the people around them don’t need to be scared of getting infected*.*” (Man*, *doctor*, *Thanh Hoa*)

These findings build upon those found in Ethiopia. An evaluation of TB infection control practices in HCW found that 38% were not following proper TB infection control practices [35]. Furthermore, this study also identified the infection control advice provided to people with TB and their family members was irregular and partial [35].

### 3.3. Drivers of self-isolation

The drivers behind self-isolation are multidimensional and interconnected. It is challenging to disentangle these drivers to discuss one factor in isolation. In summary, people with DR-TB self-isolate because they fear transmitting the disease to others, fear being stigmatised and fear self and familial reputation loss.

#### 3.3.1. Fear of infecting others

The desire for a person with DR-TB in Vietnam to self-isolate was fuelled by their fear of giving someone they know their debilitating and stigmatising condition and the anticipated stigma of transmitting DR-TB. One reason that people with DR-TB in Vietnam chose to self-isolate is to spare their family and friends from even the most remote risk of contracting DR-TB. Many patients expressed a deep-rooted fear of transmitting their DR-TB to others, which, irrespective of discussions and advice received from their doctor, could see them self-isolate until they complete treatment or beyond. The physical isolation did not only pertain to distancing, but many patients also used extra precautions such as having separate eating utensils to prevent disease transmission. A male DR-TB outpatient described that despite knowing his test results were negative for DR-TB, and his ability to transmit DR-TB was very low, he continued to isolate due to fear of transmitting DR-TB to his family:

“*I am still living with my family*, *but I sleep in a private room on a private floor*. *I also have my own bowl and chopsticks; my things are separate… I am afraid to spread [DR-TB] (hesitated)*… *it just makes me feel easy*. *The doctor said…the smear and culture results were negative…so the ability to spread it [DR-TB] is relatively low*. *However*, *I keep isolating*, *it makes me*, *and my family feel easier” (Man*, *married*, *under 40*, *Hanoi-6 months of treatment*)

#### 3.3.2. Fear of stigma

Self-isolation was also perpetuated by their fear of stigmatization and the social repercussions should a person they know become infected with TB. This fear is entangled with their fear of infecting someone they know and care about. The participant below chose to self-isolate beyond her infectious period to minimise the stigma that she might face from her community and to prevent others from experiencing DR-TB and “having a bad life like mine”. There is also an altruistic and self-sacrificial aspect to this behaviour.

*“The second challenge is the stigma of people around me… I fear that I could transmit the disease to others and that people will have stigma towards me*… *Even though I have completed the infectious period*, *I still fear that I could be the infectious source to others*, *and they will have a bad life like mine now*. *I am very sad*, *indeed (emotional)” (Woman*, *single*, *under 30*, *Hanoi-4 months of treatment*)

Ostensibly, the primary purpose of physical isolation is to prevent transmission. However, in this study we find it also plays complex social functions including reputational rehabilitation, stigma deflection, and avoidance of potential liability. If the person with DR-TB was believed to have infected someone else, they would experience guilt from giving this challenging condition to another person. The guilt would be perpetuated by their feelings of letting their community down, which is linked to the collective society, where it is everyone’s duty to protect each other [36].

While not to the extend seen in Vietnam, self-isolation, and the reasons behind it, has been described in other settings. For example, in Brazil, people with TB fear being stigmatised and transmitting the disease to their family members were found to be enablers of self-isolation [37].

#### 3.3.3. The protection of self and familial reputation

In Vietnam and many other cultures, there is considerable sociocultural pressure on individuals to behave in a socially acceptable manner. This has been described in the literature where people feel a strong pressure to protect ‘thể diện’, which roughly translates to face, honour or prestige [38]. By contracting not only TB but DR-TB, which is perceived to be worse, there is an inherent loss of ‘thể diện’. People can also experience reputational loss by being accused of transmitted it and being blamed for failing to protect their community. This can place a large burden on people with DR-TB and people with other infectious diseases to perform as self-sacrificial “perfect patients”, who behave in ways that protect ‘thể diện’. The participants described that they would become “a bad person” if they failed to do their duty of protecting others. To achieve this altruistic goal, many participants reported self-isolating as a form of self-sacrifice, that ultimately would help to restore their reputation.

*“I did not visit anyone*… *I do not want to (go anywhere) as they (other people) may have DR-TB but be in the window period*, *and if I met them*, *they would think I was the cause of transmission*… *I would get a bad reputation so I need to be careful… as I know the disease*, *I should be careful” (Man*, *married*, *under 60*, *Hanoi- 3 months of treatment*)

The following participants highlight how the three concepts, fear of infecting others, the fear of stigmatization and the management of reputation are all entangled and drivers for their self-isolation.

*“I try my best to abstain so far… I also keep away from others*. *If someone that I knew got DR-TB*, *people would suspect that I infected him or her*. *It would be very bad*, *I would blame myself for spreading disease to others*, *I will feel sad” (Man*, *married*, *under 40*, *Hanoi-6 months of treatment*)

                                 ----

*“Patients should protect others*… *In case they were infected after half an hour of being with us*, *we would feel regretful for our whole life…I should isolate myself from others*. *If somebody got infected from me*, *I would be blamed*, *I would become a bad person*.*” (Man*, *married*, *under 50*, *Hanoi-months of treatment*)

In Vietnam, and many other cultures, one’s behaviour is a reflection of not only themselves but also their immediate and extended family [39]. Reputation is also intergenerational and can influence the ‘thể diện’ of future generations. This can place pressure on family members to perform the social roles equated with good citizenship (e.g., performative isolation as a gesture of protecting others from disease). The desire to protect the ‘thể diện’ has also led to late term abortion in Vietnamese women who were conflicted with social norms, socioeconomic position and their desires regarding the pregnancy [38]. In people with DR-TB, this study identified a performative isolation where the individual would isolate longer than medically required and abstains from participation in social life for self-sacrificial and symbolic purposes. This, in conjunction with fear of being infected, led to some family members encouraging or forcing isolation of the person with DR-TB or, in extreme circumstances, exiling them from the family as mentioned in section 3.1.

### 3.4. Mitigating reputation loss and stigma

A mechanism for mitigating stigma and reputation loss due to DR-TB is selective disclosure using the term ‘lao lực’, a traditional belief for a type of TB caused by working too hard. In Vietnamese, there are four commonly used words to describe the different traditional types of TB. These include ‘lao di truyền’—hereditary TB, which is passed through a family, which as mentioned above, could lead to significant loss of reputation, ‘lao tâm’—TB caused by worrying too much which is usually associated with women and ‘lao phổi’—dangerous TB germs that are transmitted through the air. The first term, ‘lao lực’ is perceived to be less infectious and a more noble way of contracting the condition–by working hard to provide for your family [40] (Table 2).

**Table 2:** Types of tuberculosis in Vietnam, adapted from [40].

Vietnamese term	English translation	Description
Lao di truyền	Hereditary TB	Transmitted down through the generations—regardless of gender and age. Less infectious.
Lao lực	Physical TB	Caused by working too hard, poverty or poor nutrition. Less infectious. More men affected
Lao tâm	Mental TB	Caused by too much worrying, poverty, or unhappy life. Less infectious. More women affected
Lao phổi	lung TB	Dangerous and caused by TB bacteria, transmitted through the air. Associated with drinking, smoking, and eating out. More infectious. More men affected

TB = tuberculosis.

Several participants described developing TB from working too hard or by not taking care of themselves. These statements were sometimes juxtaposed with the actual source of infection. For example, the patient below described a clear understanding that she contracted the DR-TB bacteria from her husband. However, she expressed that she got TB from working too hard and not taking care of herself.

*“I underestimated the situation and worked too hard*. *I do not think it is my husband’s fault*, *it is mine because I did not take care of my health… certainly*, *I got bacteria from my husband*, *but the active situation is because of me*. *I underestimated my health and did not take care of myself” (Woman*, *married*, *under 30*, *Hanoi-3 months of treatment*)

The HCWs were aware of these terms and were able to clearly describe why some patients choose to tell others that they have ‘lao lực’ and how it can minimise the stigma they may experience. They also explained clinical TB to their patients.

*“A*: *just a few patients say so [that they have lao lực]*… *All TB is caused by bacteria and people get TB if their resistance is not good*… *People in the past did not have good nutrition and worked very hard*, *and then they got TB*. *It is called overworking TB [lao lực]*. *It is believed that that kind of TB is not infectious*… *As they [people with DR-TB] have self-stigma*, *they fear discrimination*, *so they told others that there are two types of TB*. *One is overworking TB [lao lực] and the other is genetic TB [lao di truyền] so they explained that nobody else in their family got TB with the hope that they are less discriminated… they call it overworking TB [lao lực] to make others have less discrimination*. *Many patients were advised about TB*, *but they keep telling others that they have overworking TB [lao lực]*, *not genetic TB [lao di truyền]”* (Woman, doctor, Hanoi)

In Vietnam, TB disease has multiple meanings and nomenclature that serve to attach or deflect social approbation [40]. Some of these types are shared by other cultures such Zambia which also believe there is as form of hereditary TB [41]. The various framings of TB–as a consequence of socially valued hard work or negatively viewed neglect, epitomize how a TB diagnosis can be rhetorically managed to limit the societal fallout [40].

**Fig 1:**
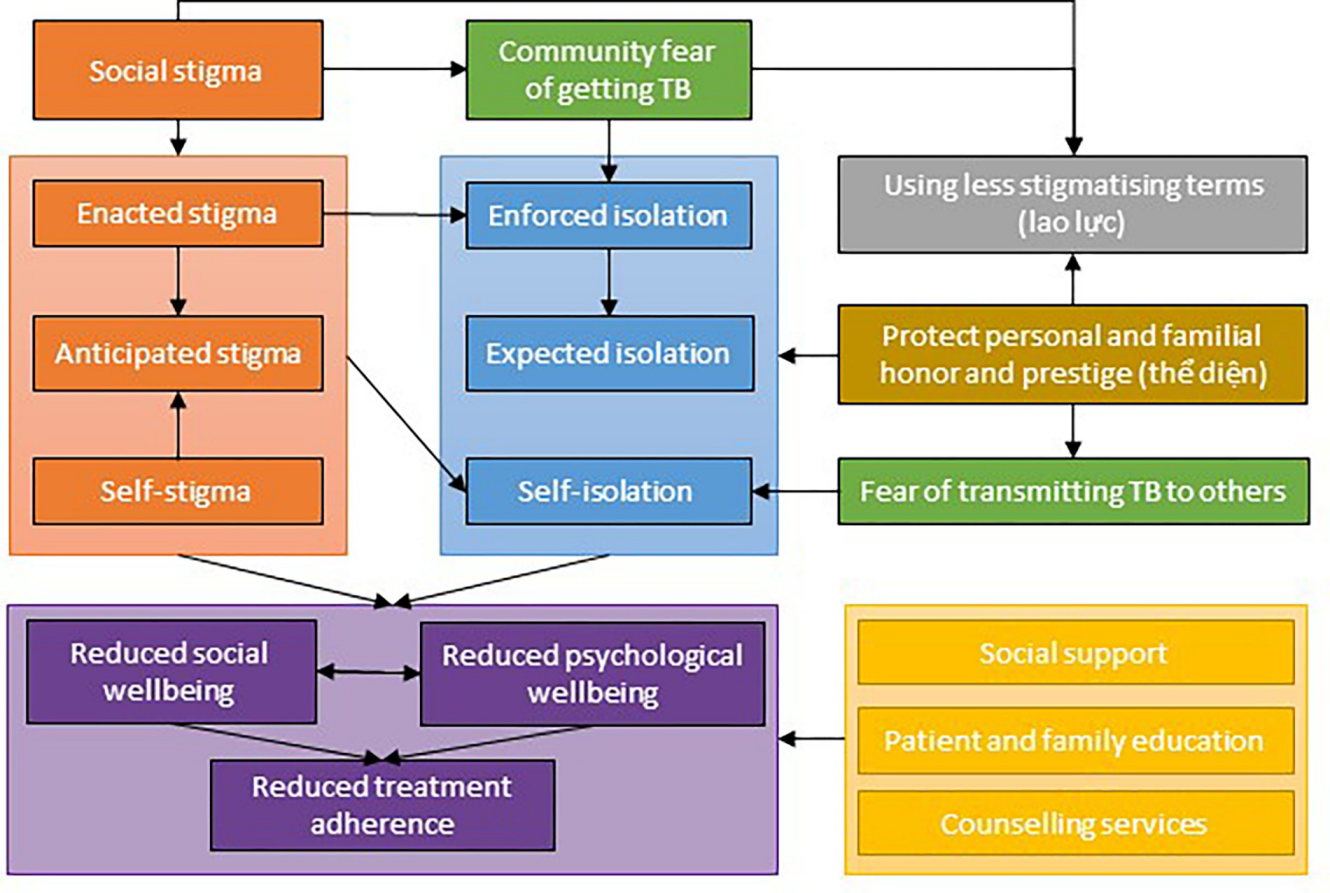
The drivers and consequences of stigma and isolation in people with DR-TB in Vietnam. DR-TB = drug resistant tuberculosis. Legend: Orange = types of stigma, blue = types of isolation, green = fears, purple = consequences of stigma and isolation, yellow = mediating factors.

In summary, this study found that despite being informed when they are no longer infection, stigma and isolation prevailed in people with DR-TB. This study identified three interconnected drivers of physical and social isolation in Vietnam including fear of infecting others, fear of stigma, and protecting their self and familial reputation or ‘thể diện’. These drivers of isolation were intertwined and often appeared alongside each other, as evidenced in the participants’ narratives. The participants described many challenges that they face and expressed a desire to prevent inflicting the disease on other people. This was compounded by the anticipated stigma associated with being blamed if they were thought to transmit DR-TB to someone they knew. This was also linked to the cultural aspect of family honour or reputation or ‘thể diện’, where people with DR-TB either self-isolated or were encouraged to withdraw from social life for longer than required by their families to be viewed as moral citizens. One method employed by participants to mitigate the stigma and potential reputation loss, alongside isolation, was to use fewer stigmatising terms for their condition, such as lao lực’ which is a less infectious and more honourable description/type of TB (Fig 1).

All these factors combined could increase the stigma and isolation of the person with DR-TB. Given the significant social consequences associated with disease transmission, it is understandable that people with DR-TB choose to isolate longer than medically necessary.

Cultural beliefs influence individual behaviours and therefore the control of infectious diseases [42,43]. Social isolation in people with DR-TB reduced their psychological wellbeing; however, collectivist cultures, such as Vietnam, may lead to public health benefits for infectious disease control. For example, the response to the recent COVID-19 pandemic saw collectivist cultures fare better with some prevention behaviours such as masking than individualist cultures, with resulted in reduced disease transmission [36,44]. Furthermore, shared cultural values can be evoked to enhance public health actions. The global TB community should strive to deepen the linkages with cultural and social values that influence patient and public actions and work with them to improve TB care.

This study highlighted the importance of engaging with local community leaders in the elimination of infectious diseases. In Vietnam and in many other contexts, isolation was an expectation, with significant social consequences if they were thought to breach this expectation and transmit DR-TB to others. These behaviours are often described as embodying a Vietnamese collectivist mentality–which emphasises loyalty, harmony, cooperation and conformity with social norms [45]. The National Tuberculosis Program needs to develop a stigma reduction strategy to enable the scale up of culturally appropriate stigma reduction and social support programs for both people with DR-TB and their families. Furthermore, HCW should receive further education to ensure that the clinical advice given to people with DR-TB is evidence-based, aligning with the social and epidemiological risks faced by patients.

Data saturation was met with a small but varied selection of participants. The study participants were in Northern Vietnam, which limits the generalisability of the findings to other regions and countries. Further research could provide specific insights into patient sub-populations such as people with HIV/DR-TB or diabetes/DR-TB coinfection.

## 4. Conclusion

This study explored stigma and isolation in people with DR-TB in Vietnam. Stigma and the drivers of isolation in people with DR-TB were intertwined and influenced by the local culture and cultural expectations. Despite being informed that they are no infectious by their HCW, people with DR-TB often chose to continue to isolate for several reasons. These findings reinforce the importance of culturally appropriate stigma reduction interventions and social support for people with DR-TB for the duration of treatment. Future research and policy updates should include culturally considered advice for patients and their family members and provide social support in the absence of physical contact.

## Supporting information

S1 Text Topic guides. (DOCX)

S2 Text Example coding guide. (DOCX)

## References

[1] World Health Organization. Tuberculosis: Key facts. 2021 [cited 3 Nov 2021]. Available: https://www.who.int/news-room/fact-sheets/detail/tuberculosis.

[2] World Health Organization (WHO). Global Tuberculosis Report. 2020.

[3] Alene KA, Clements ACA, McBryde ES, Jaramillo E, Lönnroth K, Shaweno D, et al. Mental health disorders, social stressors, and health-related quality of life in patients with multidrug-resistant tuberculosis: A systematic review and meta-analysis. J Infect. 2018;77: 357–367. doi: 10.1016/j.jinf.2018.07.007 30036607

[4] Shringarpure KS, Isaakidis P, Sagili KD, Baxi RK, Das M, Daftary A. “When treatment is more challenging than the disease”: A qualitative study of MDR-TB patient retention. PLoS One. 2016;11: 1–12. doi: 10.1371/journal.pone.0150849 26959366 PMC4784928

[5] Yin J, Wang X, Zhou L, Wei X. The relationship between social support, treatment interruption and treatment outcome in patients with multidrug-resistant tuberculosis in China: a mixed-methods study. Trop Med Int Heal. 2018;23: 668–677. doi: 10.1111/tmi.13066 29691959

[6] Morris MD, Moser K, Laniado-laborin R. Social, economic, and psychological impacts of MDR-TB treatment in Tijuana, Mexico: A patient’s perspective. Int J Tuberc Lung Dis. 2013;17: 954–960. doi: 10.5588/ijtld.12.0480 23743315 PMC3769163

[7] Baek S-B. Psychopathology of social isolation. J Exerc Rehabil. 2014;10: 143–147. doi: 10.12965/jer.140132 25061592 PMC4106767

[8] Nicholson NR. Chapter 6: Social Isolation. 9th ed. In: Larsen PD, editor. Lubkin’s Chronic Illness Impact and Intervention. 9th ed. Burlington: Jones & Bartlett Learning; 2014.

[9] Mitchell EM, van den Hof Authors Agnes Meershoek S, Zwerling A, Daftary A, Citro B, Smyth C, et al. TB Stigma Measurement Guidance. Mitchell EM., van den Hof S, editors. Challenge TB. Challenge TB; 2018. Available: www.challengetb.org.

[10] World Health Organization (WHO). Guidelines on tuberculosis infection prevention and control 2019. Who. Geneva: World Health Organisation; 2019. doi: 10.1017/CBO9781107415324.004 30933444

[11] Dharmadhikari AS, Mphahlele M, Venter K, Stoltz A, Mathebula R, Masotla T, et al. Rapid impact of effective treatment on transmission of multidrug-resistant tuberculosis. Int J Tuberc Lung Dis. 2014;18: 1019–1025. doi: 10.5588/ijtld.13.0834 25189547 PMC4692272

[12] Ritchie SR, Harrison AC, Vaughan RH, Calder L, Morris AJ. New recommendations for duration of respiratory isolation based on time to detect Mycobacterium tuberculosis in liquid culture. Eur Respir J. 2007;30: 501–507. doi: 10.1183/09031936.00131406 17537768

[13] Petersen E, Khamis F, Migliori GB, Bay JG, Marais B, Wejse C, et al. De-isolation of patients with pulmonary tuberculosis after start of treatment—clear, unequivocal guidelines are missing. Int J Infect Dis. 2017;56: 34–38. doi: 10.1016/j.ijid.2017.01.029 28163167

[14] Long NH, Johansson E, Diwan VK, Winkvist A. Fear and social isolation as consequences of tuberculosis in Vietnam: A gender analysis. Health Policy (New York). 2001;58: 69–81. doi: 10.1016/s0168-8510(01)00143-9 11518602

[15] Leigh-Hunt N, Bagguley D, Bash K, Turner V, Turnbull S, Valtorta N, et al. An overview of systematic reviews on the public health consequences of social isolation and loneliness. Public Health. 2017;152: 157–171. doi: 10.1016/j.puhe.2017.07.035 28915435

[16] Clair R, Gordon M, Kroon M, Reilly C. The effects of social isolation on well-being and life satisfaction during pandemic. Humanit Soc Sci Commun. 2021;8: 1–6. doi: 10.1057/s41599-021-00710-3

[17] Loades ME, Chatburn E, Higson-Sweeney N, Reynolds S, Shafran R, Brigden A, et al. Rapid Systematic Review: The Impact of Social Isolation and Loneliness on the Mental Health of Children and Adolescents in the Context of COVID-19. J Am Acad Child Adolesc Psychiatry. 2020;59: 1218–1239.e3. doi: 10.1016/j.jaac.2020.05.009 32504808 PMC7267797

[18] Da Silva RD, De Luna FDT, De Araújo AJ, Camêlo ELS, Bertolozzi MR, Hino P, et al. Patients’ perception regarding the influence of individual and social vulnerabilities on the adherence to tuberculosis treatment: A qualitative study. BMC Public Health. 2017;17: 1–9. doi: 10.1186/s12889-017-4752-3 28927386 PMC5606083

[19] Deshmukh RD, Dhande DJ, Sachdeva KS, Sreenivas AN, Kumar AMV, Parmar M. Social support a key factor for adherence to multidrug-resistant tuberculosis treatment. Indian J Tuberc. 2018;65: 41–47. doi: 10.1016/j.ijtb.2017.05.003 29332647

[20] Tola HH, Tol A, Shojaeizadeh D, Garmaroudi G. Tuberculosis treatment non-adherence and lost to follow up among TB patients with or without HIV in developing countries: A systematic review. Iran J Public Health. 2015;44: 1–11. 26060770 PMC4449995

[21] Attride-Stirling J. Thematic networks: an analytic tool for qualitative research. Qual Res. 2001;1: 385–405.

[22] Strauss A, Corbin J. Basics of qualitative research: Grounded theory procedures and techniques. Newbury Park, California: SAGE Publications; 1990.

[23] World Health Organization. Viet Nam Tuberculosis Profile. 2020 [cited 5 Apr 2021]. Available: https://worldhealthorg.shinyapps.io/tb_profiles/?_inputs_&entity_type=%22country%22&lan=%22EN%22&iso2=%22VN%22.

[24] Nguyen VH, Tiemersma EW, Nguyen HB, Cobelens FGJ, Finlay A, Glaziou P, et al. The second national tuberculosis prevalence survey in Vietnam. PLoS One. 2020;15: 1–15. doi: 10.1371/journal.pone.0232142 32324806 PMC7179905

[25] Palinkas LA, Horwitz SM, Green CA, Wisdom JP, Duan N, Hoagwood K. Purposeful sampling for qualitative data collection and analysis in mixed method implementation research. Adm Policy Ment Heal. 2015;42: 533–544. doi: 10.1007/s10488-013-0528-y 24193818 PMC4012002

[26] Gale N, Heath G, Cameron E, Rashid S, Redwood S. Using the framework method for the analysis of qualitative data in multi-disciplinary health research. BMC Med Res Methodol. 2013;13. 24047204 10.1186/1471-2288-13-117 PMC3848812

[27] Lincoln YS, Guba EG. Naturalistic Inquiry. Beverly Hills, California: SAGE Publications; 1985.

[28] Pescosolido BA, Martin JK. The Stigma Complex. Annu Rev Sociol. 2015;41: 87–116. doi: 10.1146/annurev-soc-071312-145702 26855471 PMC4737963

[29] Nyangoma M, Bajunirwe F, Atwine D. Non-disclosure of tuberculosis diagnosis by patients to their household members in south western Uganda. PLoS One. 2020;15: e0216689. doi: 10.1371/journal.pone.0216689 31978111 PMC6980409

[30] Bond V, Floyd S, Fenty J, Schaap A, Godfrey-Faussett P, Claassens M, et al. Secondary analysis of tuberculosis stigma data from a cluster randomised trial in Zambia and South Africa (ZAMSTAR). Int J Tuberc Lung Dis. 2017;21: 49–59. doi: 10.5588/ijtld.16.0920 29025485

[31] Alene KA, Clements ACA, McBryde ES, Jaramillo E, Lönnroth K, Shaweno D, et al. Mental health disorders, social stressors, and health-related quality of life in patients with multidrug-resistant tuberculosis: A systematic review and meta-analysis. J Infect. 2018;77: 357–367. doi: 10.1016/j.jinf.2018.07.007 30036607

[32] Tadesse S. Stigma against tuberculosis patients in Addis Ababa, Ethiopia. PLoS One. 2016;11: e0152900. doi: 10.1371/journal.pone.0152900 27054714 PMC4824500

[33] Oladimeji O, Ushie BA, Udoh EE, Oladimeji KE, Ige OM, Obasanya O, et al. Psychosocial wellbeing of patients with multidrug resistant tuberculosis voluntarily confined to long-term hospitalisation in Nigeria. BMJ Glob Heal. 2016;1: e000006. doi: 10.1136/bmjgh-2015-000006 28588950 PMC5321341

[34] Anh LTN, Kumar AMV, Ramaswamy G, Htun T, Thi TTH, Nguyen GH, et al. High levels of treatment success and zero relapse in multidrug-resistant tuberculosis patients receiving a levofloxacin-based shorter treatment regimen in Vietnam. Trop Med Infect Dis. 2020;5: 43. doi: 10.3390/tropicalmed5010043 32164231 PMC7157716

[35] Tamir K, Wasie B, Azage M. Tuberculosis infection control practices and associated factors among health care workers in health centers of West Gojjam zone, Northwest Ethiopia: A cross-sectional study. BMC Health Serv Res. 2016;16: 1–11. doi: 10.1186/s12913-016-1608-y 27503430 PMC4977729

[36] Airhihenbuwa CO, Iwelunmor J, Munodawafa D, Ford CL, Oni T, Agyemang C, et al. Culture matters in communicating the global response to COVID-19. Prev Chronic Dis. 2020;17: 1–8. doi: 10.5888/pcd17.200245 32644918 PMC7367065

[37] De Andrade EDT, Hennington ÉA, De Siqueira HR, Rolla VC, Mannarino C. Perspectives of patients, doctors and medical students at a public university hospital in rio de janeiro regarding tuberculosis and therapeutic adherence. PLoS One. 2015;10: 1–17. doi: 10.1371/journal.pone.0137572 26360291 PMC4567070

[38] Gallo MF, Nghia NC. Real life is different: A qualitative study of why women delay abortion until the second trimester in Vietnam. Soc Sci Med. 2007;64: 1812–1822. doi: 10.1016/j.socscimed.2007.02.005 17355899

[39] Kien NT. The “sacred face”: What directs Vietnamese people in interacting with others in everyday life. J Soc Sci Humanit. 2015;1: 246–259. 10.1172/vjossh.v1i3.29.

[40] Nguyen HL, Johansson E, Diwan VK, Winkvist A. Different tuberculosis in men and women: Beliefs from focus groups in Vietnam. Soc Sci Med. 1999;49: 815–822. doi: 10.1016/s0277-9536(99)00171-9 10459892

[41] Cremers AL, De Laat MM, Kapata N, Gerrets R, Klipstein-Grobusch K, Grobusch MP. Assessing the consequences of stigma for tuberculosis patients in urban Zambia. PLoS One. 2015;10: e0119861. doi: 10.1371/journal.pone.0119861 25806955 PMC4373828

[42] Clair M, Daniel C, Lamont M. Destigmatization and health: Cultural constructions and the long-term reduction of stigma. Soc Sci Med. 2016;165: 223–232. doi: 10.1016/j.socscimed.2016.03.021 27020492 PMC5758051

[43] Yang LH, Kleinman A, Link BG, Phelan JC, Lee S, Good B. Culture and stigma: Adding moral experience to stigma theory. Soc Sci Med. 2007;64: 1524–1535. doi: 10.1016/j.socscimed.2006.11.013 17188411

[44] Huang F, Ding H, Liu Z, Wu P, Zhu M, Li A, et al. How fear and collectivism influence public’s preventive intention towards COVID-19 infection: a study based on big data from the social media. BMC Public Health. 2020;20: 1–9. doi: 10.1186/s12889-020-09674-6 33198699 PMC7667474

[45] Pham VB. The Vietnamese Family in Change: The Case of the Red River Delta. Richmond: Curzon Press; 1999.

