# Supplementary material for: Good citizens, perfect patients, and family reputation: Stigma and prolonged isolation in people with drug-resistant tuberculosis in Vietnam
Source: PLOS Glob Public Health. 2022 Jun 22;2(6):e0000681. doi: 10.1371/journal.pgph.0000681 (PMC10021913; doi:10.1371/journal.pgph.0000681)
Supplement: S1 Text — (DOCX) [file pgph.0000681.s001.docx]

**Supplementary material 1: Good citizens, perfect patients, and family reputation: Stigma and prolonged isolation in people with drug-resistant tuberculosis in Vietnam**

**Topic Guide: People with DR-TB**

| **Introduction:** *Good morning/afternoon, my name is [Name of interviewer], and I will be interviewing you today. If you don’t mind, my colleague, [Name of observer], will be sitting in the room observing the interview. I will start with some general questions, then I will go into more details of your experiences as a person with DR-TB. Before we begin I would like to remind you of how grateful we are for their time and that there are no wrong or right answers. This is a time for you to share your story and your knowledge. This interview will be audio recorded. I would like to reassure you that anything they say will be treated in confidence and that your name will not be published on any documents to do with the study. You are allowed to skip questions if you feel uncomfortable or you can ask me to repeat a question if it’s not clear.* |
| --- |

| **Key area of investigation** | **Themes** | **Example questions** | **Notes** |
| --- | --- | --- | --- |
| **Basic background questions** | - Income - Education - DR-TB treatment months - Age | - Can you tell me a bit about yourself? - How long have you been taking treatment for? - What do you do for work? Do/will you go back to the same job after treatment? Why? Whose choice? |  |
| **Daily life and social circle** | - Household - Relationships - Social activities | - Can you tell me about your home? Who do you live with? - How is your relationship with your partner? Do they help to care for you? - How many children do you have? Where do they live? - What do you like to do in your spare time? With whom? |  |
| **Health facility experiences and treatment** | - How they started treatment - Treatment adherence - Health care workers - Knowledge - Personable - Side-effects | - Can you tell me a bit about your TB story? How did you get diagnosed? - Why have you decided to get treated at this facility? - What do you think about the care being provided? - How are your relationship with the health care workers? - How do you think HCWs view people with DR-TB? - How do the medications affect you? How have these side-effects affected your life? - Do you feel like the medications are working? - What were the health care workers recommendations for infection control when you are an outpatient? - Will you follow these guides? What is your and your families plan? (find out whose idea it is, and why) |  |
| **Perceived Stigma (1)** | - Interpretation to infection control - Disclosure | - When at the facility do they ask you to wear a mask? - Did you wear a mask before you got sick? How often? - How often do you wear a mask now? - How does this make you feel wearing a mask now when compared to before you got sick? - Who have you told personally that you have DR-TB? Why did you chose these people to tell? - How did they react? - Has anyone found out that you did not want to know? How did this happen? How did this make you feel? - *If previous TB patient:* Where there any differences in reactions between telling them about TB or DR-TB? - Is there anyone that you regret telling, or regret that they know you are sick? Why? |  |
| **Experienced Stigma** | - Community members - Microaggressions - Friends - Family | - Do people in your community know that you have DR-TB? How did they find out? Where did they think you were for 3 months? (while inpatient) - Has your diagnosis impacted your relationship with your family? Friends? Work colleagues? How? - Why do you think they did this/that? Is it true? - Do you think all people with TB, including people with DR-TB, are treated the same? Why/why not? - Do you get many visitors? More or less than before diagnosis |  |
| **Internalised Stigma** | - From diagnosis (intrinsic) - From experiences (extrinsic) - Coping mechanisms - Blame/shame - Mental Illness | - When you first found out your diagnosis, how did you feel, what went through your mind? - *If previous person with TB:* Was this different to your TB diagnosis? - How do you think that you got sick? - Do you ever blame yourself for getting sick? - What about your partner/others? How do they think this happened? - Do you think anyone blames you? - Is there anything in relation to your TB that you are afraid of? - Have you ever found it challenging to leave the house? - Do you ever have trouble sleeping? - Do you still feel this way? Why/ why not? What helped them to feel better? |  |
| **Perceived Stigma (2)** | - Isolation - Loss of Face | - How has DR-TB affected you? - Do you still attend your normal social events? Why/why not? - Do you feel like you have lost confidence due to your illness? How do you think this happened? - Do you feel like you are less respected by others/your family, you community now? - Have you ever tried to hide your symptoms? How did you do this? |  |
| **Coping** | - Coping - Aspirations - Hopes | - Has there been anything in your life has helped you to get through this difficult period in your life? - What do you think would have made your DR-TB treatment easier? - Do you have any plans for when you have completed treatment? Why/why not? - What would you like to happen next?/What do you think will happen? |  |
| **Closing** | - Expressing gratitude for their time so far - Final additions - Express gratitude again | - We’re nearly done, so thank you for your time in this interview. It has been really interesting and I have already learnt a lot that will be very helpful for our study. - Is there anything else that you think I should know about or would like to share with me for this research? |  |

**Topic Guide 2: Health care workers**

| **Introduction:** *Good morning/afternoon, my name is [Name of interviewer], and I will be interviewing you today. If you don’t mind, my colleague, [Name of observer], will be sitting in the room observing the interview. I will start with some general questions, then I will go into more details of your experiences working with people with DR-TB. Before we begin, I would like to remind you of how grateful we are for your time and that there are no wrong or right answers. This is a time for you to share your knowledge. This interview will be audio recorded. I would like to reassure you that anything they say will be treated in confidence and that your name will not be published on any documents to do with the study. You are allowed to skip questions if you feel uncomfortable or you can ask me to repeat a question if it’s not clear.* |
| --- |

| **Key area of investigation** | **Themes** | **Example questions** | **Notes** |
| --- | --- | --- | --- |
| **Basic background questions** | - Education - Age - Heath care experience - Household - Personal experiences with DR-TB? | - Can you tell me a bit about yourself? - What do you do for work? - Who do you live with? - Have any of your friends of family ever been diagnosed with DR-TB? |  |
| **Daily work life** | - Work structure - Challenges - Colleague relationships | - How did you come to work here? - Do you enjoy your job? - What do new staff think of this area of work? - What do you find the most challenging about working here? - Do you feel well supported in your work? - How do you cope with these challenges? - How are your relationship with the health care workers? |  |
| **Infection control** | - Policies - Practices - Fear/Blame - Colleague infection | - How does this facility prevent the spread of TB? - What mask do you wear on the DR-TB ward? - Are there sufficient infection control supplies? - Are you worried about getting DR-TB? - What recommendations do you give to the patients when they are discharged from hospital? - Has any of your colleagues ever been diagnosed with TB or DR-TB? - Explore, why, who? |  |
| **Relationship with patients** | - DR-TB clinic visits - Treatment adherence - Blame | - What care do you provide for them? - How often do you visit the DR-TB ward? - What is your relationship like with the patients? Can you give an example? - Do you have many non-adherent patients? - Why do you think they are non-adherent? - Is that a good reason - How do you and the team handle these difficult patients? - How do you think the other HCW view people with DR-TB? - Do you think anyone blames the people with DR-TB for getting so sick? |  |
| **Physical symptoms and side effects** | - DR-TB symptoms - Treatment side effects - HCW views of the symptoms and side effects - Adherence | - What symptoms do you see in your patients with DR-TB? - Do the patients ever tell you about their side effects? - What advice do you give them? - What do you think it would be like to take TB treatment? - Do you think it would be difficult? (side-effects/adherence) |  |
| **Mental**  **/emotional consequences of stigma** | - Disclosure - Blame/shame - HCW perspectives | - Why do you think people get DR-TB? - How are their relationships since being diagnosed? - Do you think people with DR-TB should tell people about their diagnosis? - How do the families and friends of people with DR-TB react to finding out about their diagnoses? - If mention blame: do you think this is true? Should people with DR-TB be blamed for their diagnosis? - How do you think having DR-TB has affected their lives? - Do you think all people with TB, including people with DR-TB, should be treated the same? Why/why not? - *If yes,* do you think they are treated the same? - Do the patients ever complain about being lonely? |  |
| **Support network** | - Support networks - Facility - Private - Social isolation - Coping mechanisms | - Are there any social services to help them? Would this be useful? (counselling) - What do you think helps keep the patients to go through this difficult treatment? |  |
| **Closing** | - Expressing gratitude for their time so far - Final additions - Express gratitude again | - We’re nearly done, so thank you for your time in this interview. It has been really interesting, and I have already learnt a lot that will be very helpful for our study. - Is there anything else that you think I should know about or would like to share with me for this research? |  |
