# Supplementary material for: Good citizens, perfect patients, and family reputation: Stigma and prolonged isolation in people with drug-resistant tuberculosis in Vietnam
Source: PLOS Glob Public Health. 2022 Jun 22;2(6):e0000681. doi: 10.1371/journal.pgph.0000681 (PMC10021913; doi:10.1371/journal.pgph.0000681)
Supplement: S2 Text — (DOCX) [file pgph.0000681.s002.docx]

| **Main Theme** | **/Sub Themes** | **Rule** |
| --- | --- | --- |
| **Self-stigma** | Disappointment | When the participant mentions feeling disappointed in life, including frustration of their condition (e.g. bored) |
|  | Blame | Any discussion about blame or fault of the patient |
|  | Fear | Discussions around fear, can include death, infecting others, disclosure or discrimination |
|  | Burden | Their actual or perceived burden on their family |
| **Family/friends** | Advice | The advice that they received regarding infection control from health care workers |
| **Community** | Relationships | Discussion of any interactions with community members or gossiping |
|  | Discriminate | Discussion of any discrimination experienced by community members |
| **Perceived stigma** | Avoiding | Avoids interactions with others for reasons other than medical/health related |
|  | Worry | When the participant states feeling uncomfortable in public as thinks others know about their diagnosis or are judging them |
| **Isolation** | Reasons | The reason that they are socially or physically isolated, including who initiated the isolation |
| **Health Care workers** | Methods | The way that the person with DR-TB was isolated, which as sleeping or eating separately, not invited to events etc. |
|  | Other | Their perception of the interactions between health care workers and other patients |
| **Infection control/advice** | Masks | Discussion of when they wear face masks, why, duration and how they feel wearing them |
| **Drivers of isolation** | **Fear of infecting others** | Discussion of the fear of infecting others, the resulting behaviours |
|  | **Fear of stigma and social repercussions** | **Fear of stigma and social repercussions** |
|  | **Reputation** | Discussions around their confidence in themselves, going about their lives and their perceived loss of reputation in their community (loss of face) |
| **Mitigating stigma** | Types of TB | Descriptions fo the different traditional types of TB in Vietnam and why they are used. |
| **Altruism/collectivist** |  | Descriptions of altruistic behaviours i.e. to protect other – personal responsibility. |
| **Disclosure** | Accounts of | Telling anyone about their diagnosis or how others found out |
|  | Reactions | How others responded to finding out about their diagnosis |
|  | Reasons for | Their rationale for why they chose to tell or not to tell other people |
| **Coping** | future | Any discussion about their future plans |
|  | Other | Discussions of other things that have helped the participant trough their MDR-TB treatment |
| **Other** |  |  |

**Supplementary material 2: Coding Framework –Patients**
